# Multiscale zonation-resolved modeling of dose-dependent determinants of acetaminophen-induced liver injury

**DOI:** 10.3389/fphar.2026.1789797

**Published:** 2026-04-21

**Authors:** Debarshi Ghosh, Stelian Camara Dit Pinto, Alon Malka-Markovitz, Mohammed Cherkaoui, Reza Sadeghi, John M. Vierling, Nicolas R. Gallo

**Affiliations:** 1 School of Digital Engineering, Computer Science and Artificial Intelligence, Long Island University, Brooklyn, NY, United States; 2 Dassault Systemes, Velizy-Villacoublay, France; 3 Department of Medicine and Surgery, Baylor College of Medicine, Houston, TX, United States

**Keywords:** APAP, liver, metabolism, multiscale, toxicity, zonation

## Abstract

**Introduction:**

Drug-induced liver injury (DILI) is a major cause of morbidity and mortality and has an important impact on drug attrition. Recent guidelines from the FDA and previous research encourage the development of virtual twin *in silico* modeling as a solution to reduce DILI impact.

**Methods:**

In this study, we used our virtual, scalable model of the human liver lobule coupled with an acetaminophen (APAP) metabolic injury model to expand the mechanistic understanding of metabolic zonation parameters involved in APAP hepatotoxicity. Using clinical overdose data, we generated a representative *in silico* patient cohort, encompassing both lower and higher APAP overdoses, and analyzed how zonal variations in metabolism factors impacted the generation of liver damage.

**Results:**

The results showed a significant difference in the sensitivity of the metabolic parameters at different overdose levels. Some, such as drug uptake rate, led to increased damage; others, such as CYP450 enzymatic activity, showed overdose-dependent effects, and others, such as the sulfation rate, showed only limited effects.

**Discussion:**

Overall, this study highlights the importance of collecting proper metabolic expression (specifically drug uptake rate, CYP450 enzymatic activity, and glutathione quantity) to ensure an accurate estimation of patient damage.

## 1 Introduction

Drug-induced liver injury (DILI) causes significant morbidity and mortality in the US and Europe (Guengerich, 2011; Hosack et al., 2023; Jaeschke and Ramachandran, 2024). Traditional animal models and early-phase clinical trials are often too slow and expensive to fully capture the range of human variability that underlies DILI or dose-dependent toxicities (Van Norman, 2020). These limitations have led to increased reliance on *in silico* approaches to complement costly experimental and clinical studies. Thus, we recently developed a scalable virtual model of the human liver lobule (Camara Dit Pinto et al., 2024). This model is based on the well-documented and well-studied acetaminophen (APAP) metabolic process of injury. This model gives us a solid base on which to showcase the study of the *in silico* approach and expand our mechanistic understanding of pathophysiology within the liver lobule. Predictive, mechanistic modeling involves the development of *in silico* patient cohorts. As a step toward *in silico* patient cohorts and as a demonstration of their capabilities, we present here a study of the impact of hepatic metabolic factors most prevalent in determining outcomes in APAP overdose.

APAP was selected because of its well-understood toxic metabolic pathway. After entering hepatocytes, approximately 90% of the APAP at therapeutic doses undergoes glucuronidation and sulfation before renal excretion, and a small fraction, approximately 5%–10%, is oxidized by the cytochrome p450 (CYP450) family of enzymes (primarily CYP1A2, CYP2E1, and CYP3A4) to form toxic N-acetyl-p-benzoquinone-imine (NAPQI) (Mazaleuskaya et al., 2015). At low APAP exposure, NAPQI is neutralized by conjugation with pre-existing glutathione (GSH) within hepatocytes (Mazaleuskaya et al., 2015). However, with higher doses of APAP, sustained NAPQI production depletes the protective GSH reserves, resulting in hepatocellular necrosis. Variations in the hepatic metabolism of APAP show differences in damage across zone 1 (periportal), zone 2 (mid-lobule), and zone 3 (centrilobular). This lobular localization of APAP-induced hepatotoxicty damage after overdoses is called zonation (Yoon et al., 2016; Yan et al., 2018; Kennedy et al., 2019). Initially, damage is localized in the centrilobular region (Hinson et al., 2010).

Mathematical models have been developed to simulate APAP metabolism and the mechanism of liver injury, but these models often lack spatial resolution or integration with systemic pharmacokinetics, limiting their translational capability. Commercial software such as DILIsym provides system-level simulation of APAP-induced liver metabolism but lacks depiction of zonation within the liver lobule (Eichenbaum et al., 2020). Other modeling approaches, such as those proposed by Remien et al. (2012), Remien et al. (2014), Reddyhoff et al. (2015), and Heldring et al. (2022), describe hepatocellular injury dynamics, but most of these models lack lobular spatial heterogeneity or simplify the heterogeneity of enzyme pathways at the lobular scale, limiting their translational use.

A multiscale approach that connects whole-body drug distribution with hepatic zonal metabolism is, therefore, essential to mechanistically link patient-specific drug exposure with clinically observed injury patterns and treatment response. The pathophysiology and zonal impact of APAP hepatotoxicity reflect a critical balance between APAP metabolism through multiple detoxification and bioactivation pathways within the liver lobule (Ramachandran and Jaeschke, 2017), along with other biological processes, such as autophagy, mitophagy, mitochondrial recovery, adaptive gene induction, inflammatory responses, repair, and other sex-dependent differences in metabolic parameters. In the present approach, we specifically focused on modeling APAP hepatic metabolism to investigate the role of metabolic zonation in the dynamics of dose-dependent APAP-induced hepatocellular injury at the lobular level.

We used our virtual, scalable model of the human liver lobule (Camara Dit Pinto et al., 2024; Malka-Markovitz et al., 2025) to expand our understanding of the metabolic zonation parameters involved in APAP-induced hepatotoxicity and showcase the abilities of *in silico* virtual cohorts.

Based on clinical overdose data, we selected four patients representing different levels of overdose. Considering those patients and a set of zonal parameter variations, an analysis was conducted to compare damage outcomes after low vs. high doses of APAP under different zonal conditions.

The results showed that at low APAP overdoses, patients with lower baseline GSH zonation were more vulnerable to hepatotoxicity. At high APAP overdoses, CYP450-mediated production of toxic NAPQI can overwhelm detoxification by GSH reserves.

## 2 Materials and methods

### 2.1 APAP overdose and clinical data

To demonstrate the *in silico* dose-dependent variability, APAP overdose clinical data were obtained from the hepatotoxic study published by Remien et al. (2012). The dataset is composed of 52 patients with severe APAP toxicity, out of which four representative cases were selected for the study:

Patient A: Low overdose: 6.1 g of APAP, 3.9 days since APAP intake.

Patient B: Medium overdose: 10.5 g of APAP, 3.4 days since APAP intake.

Patient C: Medium–high overdose: 20.5 g of APAP, 3.4 days since APAP intake.

Patient D: High overdose: 27.1 g of APAP, 4.5 days since APAP intake.

Ethics statement: Ethics consent for use of clinical patient data was not obtained since all patient data were obtained from a public domain publication of de-identified data from a protocol approved by the Institutional Review Board (IRB) of the University of Utah, in compliance with the guidelines of the Declaration of Helsinki.

### 2.2 *In silico* multiscale modeling framework

**FIGURE 1:**
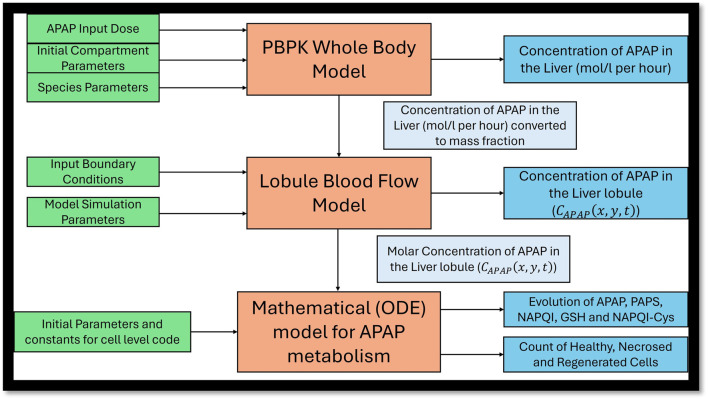
*In silico* multiscale model for APAP metabolism and liver injury prediction pipeline. Abbreviations: PBPK, physiologically based pharmacokinetic model; APAP, acetaminophen; ODE, ordinary differential equation; PAPS, 3′-phosphoadenosine 5′-phosphosulfate; NAPQI, N-acetyl-p-benzoquinone imine; GSH, glutathione; NAPQI–Cys, N-acetyl-p-benzoquinone imine–cysteine.

The *in silico* multiscale modeling framework used to showcase dose-dependent variability consists of three major components (as shown in Figure 1).

#### 2.2.1 Physiologically based pharmacokinetic (PBPK) model

To define the proper liver drug input, a representative overdose patient-specific physiologically based pharmacokinetic (PBPK) model adapted from Sluka et al. (2016) was implemented. The PBPK model implementation accurately approximated the concentration of APAP that enters the liver compartment. Unlike the earlier model, this approach accounts for the fact that APAP absorbed from the gut first enters the liver through portal venous circulation. Accordingly, the model was modified to calculate the concentration of APAP entering the liver (Equation 1):

$$C_{Liver}=\frac{Q_{Liver}\cdot C_{Art}}{V_{Liver}}+\frac{Q_{Gut}\cdot C_{PV}}{V_{Liver}},$$ (1)

where $C_{Art}$ is the arterial concentration of APAP, $C_{PV}$ is the portal venous concentration of APAP following gut absorption, $Q_{Gut}$ and $Q_{Liver}$ are the respective blood flow rates, and $V_{Liver}$ is the liver volume.

#### 2.2.2 Lobule blood flow model

The concentration of APAP derived from the PBPK model was used as an input to the lobule blood flow model, which was used to obtain the concentration of APAP available to each hepatocyte of the lobule. The blood flow dynamics to each hepatocyte of the lobule (1,705 hepatocyte cells in zone 1, 1,704 hepatocyte cells in zone 2, and 1,705 hepatocyte cells in zone 3) was simulated with healthy properties of the lobule. The blood flow dynamics simulation was performed using a transient pressure solver with the same boundary conditions and simulation parameters as described previously in our publication of a virtual scalable model of APAP hepatotoxicity prediction (Camara Dit Pinto et al., 2024). This blood flow dynamics simulation is further coupled with species transport physics and advection–diffusion physics to obtain the accurate concentration of APAP for each hepatocyte inside the liver lobule for the cellular injury mathematical model.

#### 2.2.3 APAP metabolism and hepatocyte cellular injury mathematical model (ODE)

**FIGURE 2:**
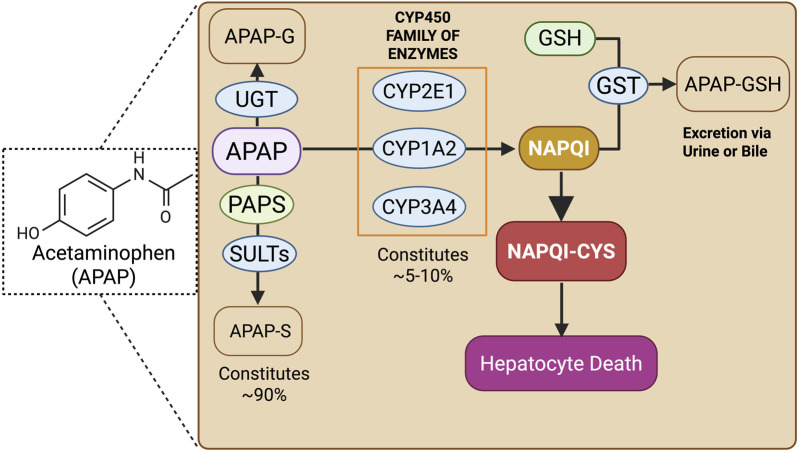
APAP cellular enzymatic metabolism and hepatocyte cellular injury. Abbreviations: APAP, acetaminophen; UGT, UDP–glucuronosyl transferases; PAPS, 3′-phosphoadenosine 5′-phosphosulfate; SULT, sulfotransferase; APAP-G, acetaminophen-glucuronidated; APAP-S, acetaminophen-sulfated; CYP450, cytochrome P450; GSH, glutathione; GST, glutathione-S-transferase; NAPQI, N-acetyl-p-benzoquinone imine; NAPQI–Cys, N-acetyl-p-benzoquinone imine–cysteine; APAP–GSH, acetaminophen–glutathione.

The cellular injury mathematical model for APAP metabolism, cellular injury, and toxicity prediction serves as an extension of our prior model, thereby more accurately depicting the effects of metabolic zonation of hepatocytes (Figure 2).

Following hepatic uptake, APAP is either sulfated (APAP-S) or glucuronidated (APAP-G), which are excreted in the urine. The process of sulfation is mediated by the enzyme sulfotransferase (SULT) and the co-substrate 3′-phosphoadenosine 5′-phosphosulfate (PAPS) (Forrest et al., 1982; Prescott, 1983; Snawder et al., 1994; Lee et al., 1996; Genter et al., 1998; Flint et al., 2017; Ramachandran and Jaeschke, 2017; Prescott, 1980). The remaining APAP is oxidized to form a toxic metabolite NAPQI, by three major isoforms of CYP450 enzymes, namely, CYP2E1, CYP1A2, and CYP3A4, as they play a major role in the oxidation of APAP to NAPQI, as evident in Araba et al. (2008); Jaeschke and Ramachandran (2024). The toxic metabolite NAPQI can cause hepatocellular injury and necrosis, unless it is detoxified by conjugation with GSH, forming APAP–GSH adducts that are excreted in the urine (Kalinec et al., 2014; Adhyapok et al., 2020). The residual toxic NAPQI binds covalently to cysteine residues in mitochondrial thiol proteins to form NAPQI–Cys adducts, which ultimately cause hepatocellular necrosis; however, a small quantity of NAPQI–Cys adducts enter the blood and are eliminated through systemic clearance and intracellular degradation (Bessems and Vermeulen, 2001; Reid et al., 2005; McGill et al., 2013; Ni et al., 2016; Dichamp et al., 2023).

Each of these enzymatic metabolism pathways was modeled separately using ordinary differential equations (ODEs), forming a coupled system to showcase the *in silico* dose-dependent variability.

The presented model explicitly includes the following:

- APAP absorption by the hepatocyte cells represented by an uptake rate constant $\left(k_{u}\right)$ that couples the extracellular APAP concentration to intracellular hepatic uptake (see Equation 2).
- Evolution of the quantity of APAP after glucuronidation, sulfation, oxidation, and reverse oxidation (see Equation 3).
- Evolution of the quantity of PAPS, which is a subproduct of the APAP sulfation reaction (see Equation 4).
- Evolution of oxidation of APAP to NAPQI by the activity of specific cytochromes from the P450 family enzymes, such as CYP1A2, CYP2E1, and CYP3A4 (see Equation 5).
- Evolution of the quantity of GSH (see Equation 6).
- Conversion of NAPQI to NAPQI–cystine adducts and its elimination through systemic clearance and intracellular degradation (see Equation 7).
- Hepatocyte health status: healthy, damaged, lysed, and regenerated (see Equations 8–11).

$$\frac{dP_{lobule}}{dt}=-\left(k_{u}\cdot P_{lobule}\right),$$ (2)

$$\frac{dP_{in}}{dt}=-\left(k_{s}*S*P_{in}\right)-\left(k_{G}*P_{in}\right)-\left(\left(k_{CYP1A2}+k_{CYP2E1}+k_{CYP3A4}\right)*\frac{P_{in}}{3}\right)+\left(k_{N}*N\right)+\left(k_{u}*P_{lobule}\right),$$ (3)

$$\frac{dS}{dt}=-\left(k_{S}*S*P_{in}\right)+b_{S}-\left(d_{S}*S\right),$$ (4)

$$\frac{dN}{dt}=\left(\left(k_{CYP1A2}+k_{CYP2E1}+k_{CYP3A4}\right)*P_{in}/3\right)-\left(k_{N}*N\right)-\left(k_{GSH}*N*G\right)-\left(k_{PSH}*N\right),$$ (5)

$$\frac{dG}{dt}=-\left(k_{GSH}*N*G\right)+b_{G}-\left(d_{G}*G\right),$$ (6)

$$\frac{dc}{dt}=\left(k_{PSH}*N\right)-\left(d_{C}*C\right),$$ (7)

$$\frac{dH}{dt}=r*H*\left(1-H+Z\right)-\eta *C*H,$$ (8)

$$\frac{dZ}{dt}=\eta *C*H-\delta_{Z}*Z,$$ (9)

$$\frac{dL}{dt}=\delta_{Z}*Z-r*H*\left(1-H+Z\right),$$ (10)

$$\frac{dR}{dt}=r*H*\left(1-H+Z\right).$$ (11)

The model parameters (as listed in Supplementary Table S1) were scaled to seconds to match the blood flow dynamics simulation, and the mathematical model was implemented in Python 3.11.7 using Spyder GUI. The coupled ODEs were solved using the SciPy package Raddu solver using the Runge–Kutta method.

#### 2.2.4 Implementation of liver metabolic zonation

**FIGURE 3:**
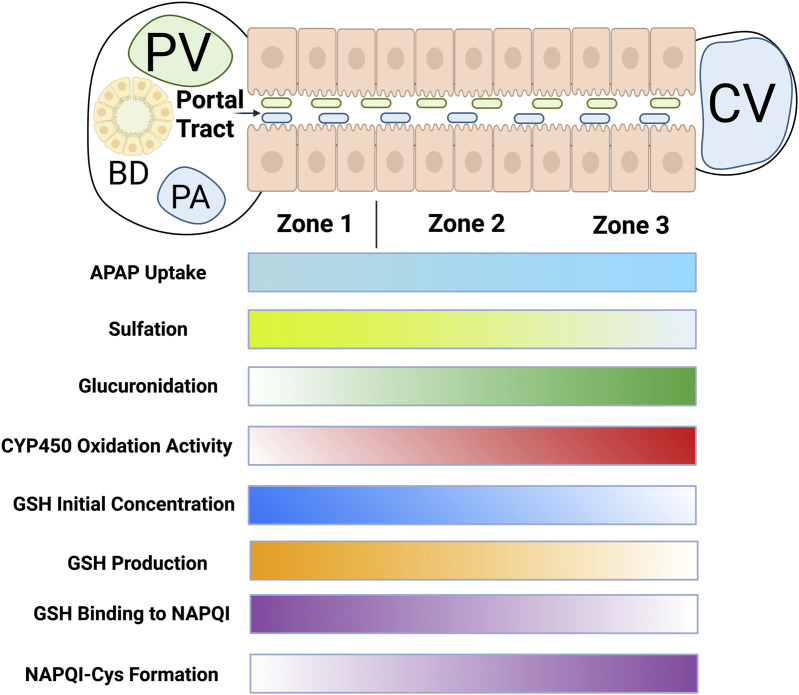
Hepatocyte zonal gradients from portal tracts to the central vein. Abbreviations: PV, portal vein; BD, bile duct; PA, portal artery; CV, central vein; GSH, glutathione; CYP, cytochrome; APAP, acetaminophen; NAPQI, N-acetyl-p-benzoquinone imine.

Liver metabolic zonation refers to the differences in the zonal organization of metabolic functions of hepatocytes from the periportal end to the central venous end of the hepatic lobule (Figure 3). The functional heterogeneity of the hepatocyte zonal gradient is clinically important and crucial for accurate modeling of *in silico* APAP dose-dependent metabolism and toxicity.

We used simulation to assess the underlying phenomena causing zonal variation in hepatocellular injury.

To do so, a number of zonal phenomena, based on existing literature evidence, were selected (see Table 1). First, the APAP uptake rate is known to be higher in the pericentral region than in the periportal region (Maeda et al., 2022). This zonation should cause a higher amount of APAP to be transported to the central region, increasing the risk of central zonal damage.

**TABLE 1:** Studied zonation phenomena.

Zonation hypothesis	Zonation phenomena	Impacted parameters	Implementation	Reference
H1	APAP uptake	$k_{u}$	Lower rate in zone 1 Higher rate in zone 3	Maeda et al. (2022)
H2	Sulfation	$k_{s}$	Lower rate in zone 3 Higher rate in zone 1	Kietzmann (2017) and Means and Ho (2019)
H3	Glucuronidation	$k_{G}$	Lower rate in zone 1 Higher rate in zone 3	Anundi et al. (1993), Kietzmann (2017), and Means and Ho (2019)
H4	CYP450 oxidation activity	$k_{CYP1A2}$ $k_{CYP2E1}$ $k_{CYP3A4}$	Lower rate in zone 1 Higher rate in zone 3	Laine et al. (2009) and Albadry et al. (2024)
H5	GSH initial concentration and production	$G_{0}$ $b_{G}$	Lower rate in zone 3 Higher rate in zone 1	Smith et al. (1979), Kietzmann (2017), and Means and Ho (2019)
H6	GSH binding to NAPQI	$k_{GSH}$	Lower rate in zone 3 Higher rate in zone 1	Kietzmann (2017) and Means and Ho (2019)
H7	NAPQI–Cys formation	$k_{PSH}$	Lower rate in zone 1 Higher rate in zone 3	Heldring et al. (2022)

Regarding the different pathways of APAP metabolism, it has been noted that the sulfation rate is higher in the periportal region than in the pericentral region, whereas the glucuronidation rate is higher in the pericentral region than in the periportal region (Kietzmann, 2017; Means and Ho, 2019). This would cause less amount of the drug to be available for transformation into NAPQI and thus cause damage in the periportal region, and correspondingly in the pericentral region. Those two phenomena were studied separately.

Regarding the oxidation of APAP, enzymatic levels and actions have been observed to be higher in the pericentral region than in the periportal region (Laine et al., 2009; Albadry et al., 2024). This would increase the amount of metabolized NAPQI, thereby increasing the chances of damage in the pericentral region.

Regarding the detoxification of NAPQI by GSH, the initial concentration and production rate have been combined into one parameter as, in the model, the initial concentration is dependent on the production rate. As observed in the literature, the rate of both of those phenomena has been observed to be higher in the periportal region (Kietzmann, 2017; Means and Ho, 2019). This zonation should show less damage in the periportal region due to the presence of a larger GSH reserve. In a similar manner, the GSH binding rate has been observed to be higher in the periportal region than in the pericentral region (Kietzmann, 2017; Means and Ho, 2019). This zonation should cause faster GSH binding to NAPQI and limit/delay the damage in the periportal area.

Finally, the binding of NAPQI with the cystine adduct has been observed to be higher in the pericentral region than in the periportal region (Heldring et al., 2022). This would cause the creation of more toxic adducts in the pericentral region, thereby causing more damage.

Those phenomena were individually studied as hypotheses, and the associated parameters from the model were altered. To study the effect of each of those parameters on zonation and define the alteration of the parameters, we introduced controlled levels of zonation for each of the zonated metabolic parameters. Four levels of zonation of ±0%, ±20%, ±50%, and ±80% were defined. For all hypotheses except H4, those levels were implemented in the model by setting a linear gradient similar to that found in Camara Dit Pinto et al. (2024). This linear gradient is calibrated between maximal and minimal values equal to the original model value plus or minus the percentile level of zonation, respectively. This choice of value ensures to maintain an average value equal to that found in the original model. The calculated values for each parameter are presented in Table 2.

**TABLE 2:** Percentage zonation of different zonation parameters.

Zonation Parameter	0% zonation	20% zonation	50% zonation	80% zonation
APAP uptake (H1)	Central: 8.33e−05	Central: 1.00e−04	Central: 1.25e−04	Central: 1.50e−04
	Portal: 8.33e−05	Portal: 6.67e−05	Portal: 4.17e−05	Portal: 1.67e−05
Sulfation (H2)	Central: 2.62e09	Central: 2.09e09	Central: 1.31e09	Central: 5.23e08
	Portal: 2.62e09	Portal: 3.14e09	Portal: 3.92e09	Portal: 4.71e09
Glucuronidation (H3)	Central: 3.64e−05	Central: 4.15e−05	Central: 5.19e−05	Central: 6.23e−05
	Portal: 3.46e−05	Portal: 2.77e−05	Portal: 1.73e−05	Portal: 6.92e−06
GSH initial concentration (H5)	Central: 6.87e−15	Central: 5.50e−15	Central: 3.44e−15	Central: 1.37e−15
	Portal: 6.87e−15	Portal: 8.24e−15	Portal: 1.03e−14	Portal: 1.24e−14
GSH production (H5)	Central: 1.59e−19	Central: 1.27e−19	Central: 7.95e−20	Central: 3.18e−20
	Portal: 1.59e−19	Portal: 1.91e−19	Portal: 2.39e−19	Portal: 2.86e−19
GSH binding (H6)	Central: 1.85e13	Central: 1.48e13	Central: 9.26e12	Central: 3.70e12
	Portal: 1.85e13	Portal: 2.22e13	Portal: 2.78e13	Portal: 3.33e13
NAPQI–Cys formation (H7)	Central: 1.27e−03	Central: 1.53e−03	Central: 1.91e−03	Central: 2.29e−03
	Portal: 1.27e−03	Portal: 1.02e−03	Portal: 6.37e−04	Portal: 2.55e−04

For hypothesis 4, the zonation of the CYP450 isoenzyme oxidation rate, because the published literature (Laine et al., 2009; Albadry et al., 2024) shows more specific zonation patterns, a different gradient of zonation following the different zonation level was applied. Of the multiple CYP450 isoenzymes that metabolize APAP, three main isoenzymes were considered (CYP2E1, CYP1A2, and CYP3A4) (Albadry et al., 2022).

As observed in the literature, using normalized enzymatic activities from liquid chromatography/mass spectroscopy (LC/MS) (Laine et al., 2009), we defined new non-zonated baseline parameters for each isoenzyme considered (see Table 3).

**TABLE 3:** Percentage zonation of the CYP450 family of enzymes.

Zonation parameter	0% zonation	20% zonation	50% zonation	80% zonation
CYP1A2 (H4)	Central: 2.60e−06	Central: 2.87e−06	Central: 3.20e−06	Central: 3.54e−06
	Portal: 2.60e−06	Portal: 2.34e−06	Portal: 1.88e−06	Portal: 1.43e−06
CYP2E1 (H4)	Central: 2.80e−06	Central: 2.86e−06	Central: 2.99e−06	Central: 3.12e−06
	Portal: 2.80e−06	Portal: 2.28e−06	Portal: 1.53e−06	Portal: 7.80e−07
CYP3A4 (H4)	Central: 5.60e−6	Central: 6.30e−06	Central: 7.43e−06	Central: 8.55e−06
	Portal: 5.60e−06	Portal: 4.55e−06	Portal: 3.06e−06	Portal: 1.56e−06

As more evidence is found for a non-linear zonation pattern of the isoenzymes in the literature, the zonation of these isoenzymes was modeled using immunohistochemistry-stained images of the human liver showing zonal expression. To keep in accordance with other parameters’ zonation levels, the non-linear patterns were defined to ensure ±0%, ±20%, ±50%, and ±80% variation from baseline, all in conservation of an average parameter equal to the original model value (as seen in Supplementary Figure S1). Simulations were run for every hypothesis and zonation level to predict the consequences on zonal hepatocellular injury.

#### 2.2.5 Sensitivity and statistical analyses

Sensitivity and quantitative analyses were performed to quantify the effect of zonated parameters on pericentral necrosis. Predicted hepatocyte state distributions (healthy, necrotic, and regenerated) were compared between zonated and non-zonated models using chi-square tests. This test serves as a statistical hypothesis testing of categorical variables such as the number of healthy, necrotic, and regenerated cells. On all results, a p-value less than 0.05 was considered statistically significant. This analysis validated that the magnitude of zonation used in this study enabled us to identify the dominant determinants of spatial zonal injury heterogeneity across the spectrum of APAP overdoses.

## 3 Results

The *in silico* multiscale modeling framework coupled with systemic pharmacokinetics was used to investigate the role of hepatocyte metabolic zonation in the dynamics of dose-dependent APAP-induced hepatocellular injury at the lobular level. A total of 112 simulations were performed to study the impact of seven zonation hypotheses across a spectrum of APAP doses. For each simulation, the time of maximum overall hepatocellular necrosis was chosen for the study. At this selected time, the hepatocytes in each state (heathy, damaged, and regenerating) were determined for each of the three lobular zones (see Supplementary Table S1). The results of simulations of the metabolic zonation levels are described in the subsections given below, comparing lower vs. higher APAP doses at ±80% zonation gradient.

### 3.1 APAP uptake rate variation

**FIGURE 4:**
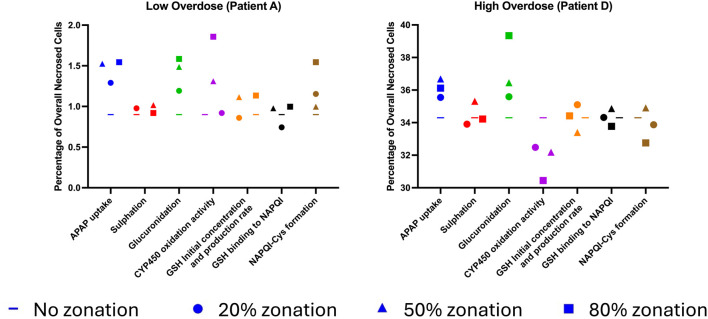
Comparative analysis of variation in the number of percentages of overall necrosed cells for different enzymatic zonation levels for a low- and high-overdose patient.

**FIGURE 5:**
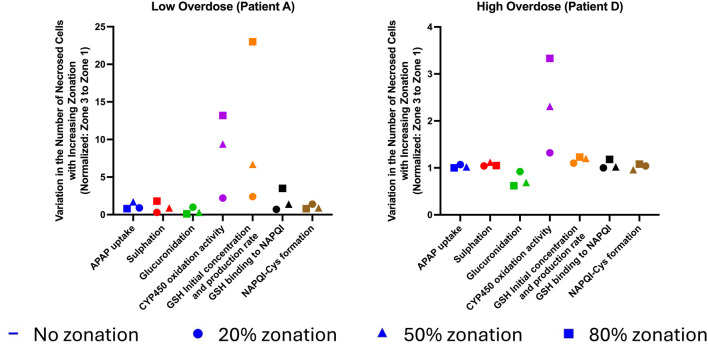
Comparative analysis of variation in the number of necrosed cells for different enzymatic zonation levels for a low- and high-overdose patient.

With respect to varying the APAP uptake rate, the results show significant changes in the damage distribution for patients A, B, and D. However, for patient C, the distribution is only statistically significant at the low zonation level (see Table 4). In the low-overdose patient (A), the maximum number of necrosed cells was 46 out of 5,114 (0.89%) without zonation and increased to 79 when 80% zonation was applied (see Figure 4). In comparison, in the high-overdose patient (D), the maximum number of necrosed cells was 1,754 out of 5,114 (34.29%) without zonation and increased to 1,876 upon applying 50% zonation (see Figure 4). Results obtained for the implementation of zonation in the APAP uptake rate showed an expected significant increase (***, p≤0.001 ) in damage in the pericentral region compared to that in the periportal region for all patients at all zonation levels (see Table 5). Zonation in the APAP uptake rate caused a maximum increase in pericentral necrosis at the intermediate (50%) zonal gradient [1.7 times for low-overdose patient (A) and 1.02 times for high-overdose patient (D)] (see Figure 5). For patient A, 32 necrosed cells (1.88%) were counted, compared to 629 (36.89%) for patient D, indicating higher drug accumulation near the central vein.

**TABLE 4:** Chi-square statistical comparison of overall necrosed cells between no zonation and zonation for different enzymatic zonation levels and overdose spectrum.

Patient	A			B			C			D		
Zonation	20	50	80	20	50	80	20	50	80	20	50	80
APAP uptake	**	***	***	*	***	***	***	​	​	**	***	*
Sulfation	​	*	​	***	​	​	​	**	*	​	​	*
Glucuronidation	​	***	***	***	***	***	*	***	***	*	**	***
CYP450 oxidation activity	​	**	***	​	​	*	***	**	***	**	***	***
GSH initial concentration and production rate	​	​	​	**	​	*	​	***	​	*	​	​
GSH binding to NAPQI	​	​	​	​	*	​	​	​	​	​	​	​
NAPQI–Cys formation	​	​	***	​	​	**	​	​	**	​	​	*

*** P-value <0.001, ** P-value <0.01, and * P-value <0.05.

**TABLE 5:** Chi-square statistical comparison in the number of necrosed cells between no zonation and zonation for different zonation hypotheses and spectrum of overdoses.

Patient	A			B			C			D		
Zonation	20	50	80	20	50	80	20	50	80	20	50	80
APAP uptake	**	***	***	*	***	***	***	***	*	**	***	**
Sulfation	***	***	​	***	​	​	​	*	**	​	**	*
Glucuronidation	​	***	***	***	***	***	*	***	***	**	***	***
CYP450 oxidation activity	***	***	***	***	***	***	***	***	***	***	***	***
GSH initial concentration and production rate	***	***	***	***	***	***	***	***	***	***	***	***
GSH binding to NAPQI	**	***	***	*	​	​	​	​	​	​	​	***
NAPQI–Cys formation	**	*	***	*	​	***	*	**	**	​	​	​

*** P-value <0.001, ** P-value <0.01, and * P-value <0.05.

### 3.2 Sulfation rate variation

Variation in the sulfation rate resulted in no significant pattern of impact of damage distribution overall (see Table 4). This suggests that variation in the sulfation rate does not significantly impact the overall damage distribution in the lobule. In the low-overdose patient (A), the maximum number of necrosed cells was 46 out of 5,114 (0.89%) without zonation and increased to 52 upon applying 50% zonation (see Figure 4). In comparison, in the high-overdose patient (D), the maximum number of necrosed cells was 1,754 out of 5,114 (34.29%) without zonation and increased to 1,806 when 50% zonation was applied (see Figure 4). Similarly to the overall damage, no significant pattern was found in the impact of the parameter on the zonation of damage (see Table 5). Sulfation rate zonation shifted necrosis to the pericentral region (see Figure 5) by 1.8 times in the low-overdose patient (A) (0.47% at 0% zonation to 1.23% at 80% zonation) and approximately 1.2 times in the high-overdose patient (D) (32.96% at 0% zonation to 37.01% at 50% zonation).

### 3.3 Glucuronidation rate variation

Based on the results obtained while varying the glucuronidation rate, zonation can be considered to have a significant impact on the overall damage distribution, with exception to the low-level zonation (20%) on the low-level overdose patient (Patient A) (see Table 4). In the low-overdose patient (A), the maximum number of necrosed cells was 46 out of 5,114 (0.89%) without zonation, which increased to 81 upon applying 80% zonation (see Figure 4). In comparison, in the high-overdose patient (D), the maximum number of necrosed cells was 1,754 out of 5,114 cells (34.29%) without zonation, which increased to 2,012 upon applying 80% zonation (see Figure 4). Similarly to the impact on the overall damage, variation in glucuronidation rate zonation showed a significant impact on the difference between the distribution in zone 1 and zone 3 for all patients and zonation levels, with exception of the low-overdose patient (Patient A) at low overdose level (20%) (see Table 5). Glucuronidation rate zonation (see Figure 5) consistently shifted the injury patterns toward periportal regions [0.3 times for the low-overdose patient (A) and 0.69 times for the high-overdose patient (D)], increasing necrosis in zone 1 and reducing it in zone 3 (from 1.11% at 0% zonation to 0.23% at 80% zonation for patient A; from 32.67% at 0% zonation to 29.03% at 80% zonation for patient D).

### 3.4 CYP450 isoenzyme rates

Based on the results obtained while varying the CYP 450 family isoenzyme activity rate, the parameter shows a consistent significant impact at the high zonation level across all patients but only a consistent impact at higher overdose for the lower zonation level (see Table 4). In the low-overdose patient (A), the maximum number of necrosed cells was 46 of 5,114 (0.89%) without zonation, which increased to 95 upon applying 80% zonation (see Figure 4). In comparison, in the high-overdose patient (D), the maximum number of necrosed cells was 1,754 of 5,114 cells (34.29%) without zonation, which decreased to 1,557 necrosed cells when 80% zonation was applied (see Figure 4). Zonation of CYP450 activity strongly influenced necrosis zonation across all patients and zonation levels (see Figure 5). In the low-overdose patient (A), increasing zonation (20%–80%) increased pericentral necrosis (by 13.2 times), with the number of necrosed cells increasing from 22 (1.29%) at 0% to 66 (3.87%) at 80% zonation, whereas in patient D (high overdose), increasing CYP450 zonation (20%–80%) increased pericentral necrosis (by 3.33 times) from 36.25% (618 necrosed cells) to over 48% (831 necrosed cells), leaving only over 37% of healthy cells.

### 3.5 GSH initial concentration and production rate variation

Based on the results obtained while varying the GSH initial concentration and production rate, no significant pattern of the impact of damage distribution overall was observed (see Table 4). In the low-overdose patient (A), the maximum number of necrosed cells was 46 out of 5,114 cells (0.89%) without zonation, which increased to 58 upon applying 80% zonation (see Figure 4). In comparison, in the high-overdose patient (D), the maximum number of necrosed cells was 1,754 out of 5,114 cells (34.29%) without zonation, which increased to 1,760 when 80% zonation was applied (see Figure 4). Results obtained for the implementation of zonation in initial GSH concentration and production rate showed an expected significant increase (***, p≤0.001 ) in damage in the pericentral region compared to the periportal region for all patients at all zonation levels (see Table 5). In the low-overdose patient (patient A), in the periportal region, more than 96% of the cells remained healthy compared to more than 98% without zonation. As expected, it increased pericentral vulnerability, with the number of necrosed cells being 23 times higher (see Figure 5), increasing from 17 necrosed cells (0.11%) without zonation to 46 necrosed cells (2.7%) with 80% zonation. In contrast, under high-overdose conditions (patient D), pericentral vulnerability increased by 1.23 times. With increasing zonation (20%–80%), pericentral necrosis increased from 562 (32.96%) to 627 (36.8%), and slightly less than 50% of the cells were healthy in the pericentral region.

### 3.6 GSH binding rate variation

Based on the results obtained while varying the GSH binding rate, no significant pattern of the impact of damage distribution overall was observed (see Table 4). In the low-overdose patient (A), the maximum number of necrosed cells was 46 out of 5,114 (0.89%) without zonation, which increased to 51 when applying 80% zonation (see Figure 4). In comparison, in the high-overdose patient (D), the maximum number of necrosed cells was 1,754 out of 5,114 (34.29%) without zonation, which increased to 1,783 when applying 50% zonation (see Figure 4). Similarly to the overall damage, no pattern of significance was found in the impact of this parameter to the zonation of damage (see Table 5). Zonation of GSH binding moderately increased [by 3.5 times for the low-overdose patient (A) and by 1.18 times for the high-overdose patient (D)] pericentral necrosis at 80% zonation (1.64% (28/1,705) for patient A and 35.72% (609/1,705) for patient D (see Figure 5).

### 3.7 NAPQI–Cys formation rate variation

Based on the results obtained while varying the NAPQI–Cys formation rate, a consistent significant impact was observed at the high zonation level (80%) across all patients. In the low-overdose patient (A), the maximum number of necrosed cells was 46 of 5,114 cells (0.89%) without zonation, which increased to 79 necrosed cells when applying 80% zonation (see Figure 4). In comparison, in the high-overdose patient (D), the maximum number of necrosed cells was 1,754 of 5,114 cells (34.29%) without zonation, which increased to 1,785 when applying 50% zonation (see Figure 4). Results obtained for the implementation of zonation in the NAPQI–Cys formation rate showed a significant effect of zonation only on lower-overdose patients but not on the highest-overdose patient (see Table 5). The NAPQI–Cys formation rate showed dose-dependent effects, enhancing pericentral necrosis (by 0.8 times) at low overdoses (1.23% at 0% zonation to 1.47% at 80% zonation).

### 3.8 Effects of metabolic zonation on overall lobular damage at lower and higher APAP overdoses

This study enabled us to perform a comparative investigation the effect of hepatocyte metabolic zonation on the overall necrosis or damage of hepatocyte cells at the lobular level across low and high overdoses of APAP (Figure 4). On increasing the zonation level, as the zonation gradient maintained a similar average parameter, we observed a similar overall amount of maximum necrosed cells. However, the results show differences in the impact of zonation for different parameters.

In the low-overdose patient (patient A), the overall necrosis remained low, approximately ranging from 0.75% to 1.85%. However, applying increased zonation showed variation in the number of overall damages for the APAP uptake rate, glucuronidation, CYP 450 enzymatic activity, and NAPQI–Cys formation rate. These parameters show a higher impact when applied to the model. The APAP uptake rate showed a clear increase as soon as zonation was applied, but a low variation with the application of different levels increasing from 1.29% at 20% zonation to 1.54% at 80% zonation. Glucuronidation followed a similar pattern as APAP uptake rate, with a direct increase in effect upon application of zonation but a low impact from increasing zonation levels. CYP 450 enzymatic activity zonation showed an increasing effect on overall damage with increase in zonation level, with effects close to baseline at low zonation and the highest increase at 80% zonation (1.85% necrosed cells). Finally, NAPQI–Cys formation rate shows slightly higher damage at 20% zonation (1.15% necrosed cells) than at 50% zonation (0.99% necrosed cells), with the highest damage observed at 80% zonation (1.54% necrosed cells). The other zonation parameters (sulfation rate, GSH initial concentration and production rate, and GSH binding rate) show a comparatively minimal effect on the overall necrosed cells. On the contrary, parameters such as GSH initial concentration and production rate, along with GSH binding rate, reduce damage at the lowest level of zonation (20%).

In the high-overdose patient (Patient D), the overall necrosis remained high, ranging from 30.4% to 39.3%. Interestingly, in the high-overdose patient (patient D), parameters show different behavior than those observed in the low-overdose patient. The APAP uptake rate and glucuronidation still showed the highest increase in overall damage when applying increasing levels of zonation. This effect was observed from the first application of the lowest level of zonation (20%). Whereas increasing zonation level showed little variation in effect for APAP uptake (35.54% at 20% zonation to 36.1% at 80% zonation), glucuronidation exhibited the greatest increase, going from 35.58% at 20% zonation to 39.3% at 80% zonation. However, on the contrary, CYP 450 enzymatic activity zonation showed a systematic decrease in overall damage. Baseline damage without zonation showed 34.3% damage overall, but applying zonation led it to decrease to 30.4% at 80% zonation. Regarding the other zonation parameters (sulfation rate, GSH initial concentration and production rate, GSH binding rate, and NAPQI–Cys formation rate), the results showed no consistent variation with an increase and decrease in damage depending on the level of zonation. However, overall, the highest level of zonation (80%) seemed to decrease the overall damage.

### 3.9 Effects of metabolic zonation levels on zonal damage at lower and higher APAP overdoses

The study enabled us to perform a comparative investigation of the effects of hepatocyte metabolic zonation on the damage gradient between periportal and pericentral zones across low and high overdoses of APAP (Figure 5). As expected, the implementation of zonation created a difference in damage between periportal and pericentral zones. However, parameters had different intensities of effects.

In the low-overdose patient (patient A), increased zonation resulted in a variation in the number of zonal necrosed cells for GSH initial concentration and production rate, CYP450 oxidation activity, GSH binding to NAPQI, and sulfation rate. GSH initial concentration and production rate were shown to increase the variation of zonal necrosed cells, with the normalized number of necrosed cells increasing from 2.4 times at 20% zonation to 23.0 times at 80% zonation. CYP450 oxidation activity followed a similar pattern as GSH initial concentration and production rate, with the normalized number of zonal necrosed cells increasing from 2.2 times at 20% zonation to 13.2 times at 80% zonation. GSH binding to NAPQI also increased the number of necrosed cells, with the variation in the number of zonal necrosed cells increasing from 0.7 times at 20% zonation to 3.5 times at 80% zonation. Finally, the sulfation rate also increased the number of zonal necrosed cells, with the variation in the number of necrosed cells increasing from 0.9 times at 20% zonation to 1.8 times at 80% zonation. The other zonation parameters (APAP uptake rate, glucuronidation rate, and NAPQI–Cys formation) show comparatively minimal effect on the variation in the number of zonal necrosed cells.

In the high-overdose patient (Patient D), applying increased zonation showed a variation in the number of zonal necrosed cells for CYP450 oxidation activity and glucuronidation rate. CYP450 oxidation activity increased the variation in zonal necrosed cells, with the normalized number of necrosed cells increasing from 1.32 times at 20% zonation to 3.33 times at 80% zonation. GSH initial concentration and production followed a similar pattern as CYP oxidation activity, with the normalized number of zonal necrosed cells increasing from 1.10 times at 20% zonation to 1.23 times at 80% zonation. On the contrary, the glucuronidation rate showed a reduction in the variation in the number of zonal necrosed cells from 0.92 times at 20% zonation to 0.62 times at 80% zonation. The other zonation parameters (APAP uptake, sulfation rate, GSH initial concentration and production, GSH binding rate, and NAPQI–Cys formation) showed a comparatively minimal effect on the zonal distribution of necrosis.

## 4 Discussion

This study presents the impact of hepatocyte metabolic zonation on the dynamics of dose-dependent APAP-induced hepatocellular injury at the lobular level through the use of *in silico* multiscale modeling. Inclusion of metabolic zonation in the model reproduced the clinically observed patterns of pericentral necrosis, validating its physiological relevance and translational utility (Kietzmann, 2017; Means and Ho, 2019). A significant contribution of this study is the incorporation of hypothesis-based incremental metabolic zonation pattern (20%, 50%, and 80%) and the analysis of its effect on cellular injury outcomes, i.e., cell necrosis. This enables us to quantitatively assess the relationship between the extent of zonation and the severity of damage, including a sensitivity analysis across varying APAP intake dosages.

The results showed an overall increase in necrosis damage when applying zonation for multiple phenomena. This increase was unexpected as all zonated parameters were specifically defined to maintain the average value similar to the non-zonated model. However, this indicates the presence of an underlying damage phenomenon linked to zonation. This shows the importance of zonation modeling to predict accurate lobular damage.

More specifically, variation in the APAP uptake rate showed a consistent increase in the overall damage distribution for patients A (low overdose), B (medium overdose), and D (high overdose) but showed a lower impact once zonation was applied. This shows the high importance of the said parameter on overall damage but low sensitivity to the zonation level. This low effect of zonation level from this parameter might be explained by the effect of intake being impactful on the overall metabolism cycle. As more APAP was ingested in the cells, other nontoxic pathways were also considerably activated, regulating the overall effect of this parameter.

Similar to the APAP uptake rate, variation in the glucuronidation rate also increased the overall damage distribution for patients A (low overdose), B (medium overdose), C (medium–high overdose), and D (high overdose). However, different from the uptake rate, the results show higher sensitivity to zonation level. This higher sensitivity can be explained by higher glucuronidation being modeled as a direct linear impact on the consumption of APAP. Thus, glucuronidation rate is highly important when modeling patient-specific damages. It is also important to note that glucuronidation, opposite to all other phenomena, naturally promotes periportal damage, making this phenomenon contrariant to clinical observation regarding necrosis zonation.

Another critical parameter in overall necrosis is CYP450 oxidation activity by isoenzymes such as CYP1A2, CYP2E1, and CYP3A4. This parameter zonation variations also showed significant effects on the overall extent of necrosis. However, the effect of this parameter is different for low and high overdoses. At low overdose, CYP450 oxidation activity shows a high impact and correlation with the level of zonation, significantly increasing the extent of necrosis with higher levels of zonation. In high-overdose patients, the effect becomes inverted and actually lowers the overall necrosis in the lobule. This can be considered a plateau effect of the enzymes. When excessive drug is absorbed, necrosis occurs quickly and triggers faster regeneration, limiting the overall necrosis in the most active zones (zone 3). However, this interpretation is only limited to the behavior of the presented model. Increasing APAP doses increases the area of necrosis (Bhushan et al., 2014), and regeneration also depends on the area of necrosis (Nguyen et al., 2022). Increased necrosis can also trigger senescence in periportal hepatocytes, which further inhibits regeneration (Umbaugh et al., 2024). Nevertheless, the different effects of CYP450 oxidation activity by isoenzymes observed under low- and high-overdose conditions make it a critical parameter for patient calibration.

Finally, variation in the NAPQI–Cys formation rate also showed significant effects on overall damage, but only at higher-level zonation. This shows a low variability of the parameter.

The other zonation parameters did not show a consistent pattern of increasing or decreasing the damage overall. This shows a low impact of those parameters and a lesser importance in the zonation effect for patient-specific modeling.

The findings show that for lower APAP overdoses (Patient A), zonal variation in the GSH initial concentration and GSH production rate strongly increases the difference in zonal, amplifying the cellular damage difference by up to 23-fold when a high (80%) zonation gradient is implemented. However, at a higher overdose, the parameter barely created a change in zonal damage. This highlights the effect of cellular GSH storage. Indeed, with higher overdose, the amount of GSH in the cell will deplete faster and would not be able to protect the cells. Therefore, changes in the amount would not greatly affect the overall results. However, at lower overdose, the chances of GSH being able to sustain and prevent injuries are higher, thus producing a higher impact.

Similarly, the gradient of CYP450 activity showed a significant impact on the difference in damage between periportal and pericentral zones. This high effect of the parameter is also consistent along low and high overdoses. This phenomenon can be amplified by the nonlinear implementation of the zonation effects. This still highlights the importance of the parameter in damage prediction and makes it the primary validated hypothesis for clinically observed zonation. Out of all the parameters, glucuronidation unexpectedly showed inverted zonation phenomenon, creating more damage in zone 1 than in zone 3 (by 0.62 times). However, its effect was low at low overdose and did not increase much at high overdose. This inverted parameter would still be important during patient model calibration as it would impact the zonation of the results. The other zonation parameters (APAP uptake rate, sulfation, GSH binding rate, and NAPQI–Cys formation) showed a comparatively minimal effect on the variation in the number of zonal necrosed cells.

Overall, this study enabled us to systematically investigate the link between hepatocyte metabolic zonation intensity (distribution between the periportal region and the pericentral region) and overall cellular necrosis across a spectrum of APAP overdose cases, specifically for low overdoses and high overdoses. The results revealed an overall greater effect of CYP450 enzyme activities, making it a driving factor of zonation and one of the primary concerns in patient-specific modeling. This study also highlights the importance of detoxification-based treatment (e.g., NAC treatment) for overdose patients and their prevalence on low-overdose patients (Licata et al., 2022).

Despite its strengths, the current model has limitations. To model the metabolic zonation pattern, the linear zonation pattern has been used as exact distribution profiles of metabolic parameters such as GSH are not known. Implementing a more precise zonation pattern (such as that done for CYP450 enzymes) would significantly enhance the prediction capability of the model. On the same idea, this study only proposed an individual study and sensitivity analysis of the parameters. If this enables us to identify the exact impact of parameters, it does not provide insights into their combined effects. Future refinement of the model with a more accurate physiological zonation distribution would likely improve the biological coherence and predictive capability of the model.

A limitation of the current APAP metabolism and hepatocyte cellular injury mathematical model is that the metabolic pathways were modeled using the predefined linear or mass action kinetics-based rate terms. Although this modeling enabled us to effectively analyze the effects of metabolic zonation, it did not fully capture the dose-dependent saturation of the metabolic enzymes. Thus, future refinement of the model with non-linear reaction kinetics could improve the quantitative prediction capability of the model, specifically at higher overdose. In addition, integrating the Michaelis–Menten rate constant equations for the cysteine adducts and its related pathways is a necessary step toward capturing the dose-dependent metabolic pathway shifts.

Additionally, the metabolic model excluded the effects of other cells in liver lobules such as inflammatory cells and its responses, which have a significant influence on the liver injury outcomes. Addressing these factors in future work can enhance the model’s prediction capabilities.

Another limitation of the current model is that it only focuses on predicted hepatocyte cell state distribution and lobular necrosis pattern using four representative overdose cases, rather than validation of the model using clinical biomarkers, such as alanine aminotransferase (ALT), aspartate aminotransferase (AST), or the international normalized ratio (INR). Future refinement, by validation of the model using clinical biomarker measurements, would further potentially improve the translational and predictive capability of the model.

Future research efforts include validation and refinement to account for the current linear metabolism and the non-refined parameter range used for the study. A more relevant and strongly validated model will enable a better estimation of the damage and more precise refinement of the metabolic effects.

More refined parameter quantification of the different metabolic phenomena (GSH levels, CYP450 enzymatic activity, and others) will allow for the development of more precise estimations from the model.

Finally, the presented research paved the way for improved understanding of the effects of spatial heterogeneity in APAP-induced liver injury. This same strategy can be extended to other drug metabolic models to explore drug-induced liver injury.

By presenting different zonation levels and metabolic activity ranges, this study presented the first step for the implementation of virtual cohorts. Applying the current methodology to a newly developed cellular metabolic drug model can be extended to whole-organ damage prediction. Additionally, using representative population metabolic specificities will pave the way for development of virtual cohorts and *in silico* drug testing, as supported by the recent FDA initiative (FDA, 2004).

## Data Availability

The original contributions presented in the study are included in the article/Supplementary Material, further inquiries can be directed to the corresponding author.
