# Supplementary material for: Multiscale zonation-resolved modeling of dose-dependent determinants of acetaminophen-induced liver injury
Source: Front Pharmacol. 2026 Apr 21;17:1789797. doi: 10.3389/fphar.2026.1789797 (PMC13139076; doi:10.3389/fphar.2026.1789797)
Supplement: Supplementary file 1 [file Supplementaryfile1.docx]

Supplementary Material

# Supplementary Data

Supplementary data can be found in the file named “Supplementary_Datasheet.xlsx”. This supplementary file represents the full results of the study.

# Supplementary Tables

*Supplementary Table 1: Model equation parameters for the cellular mathematical model with cellular enzymatic metabolism and hepatocyte cellular injury*

| **Variable** | **Symbol** | **Value** | **Unit** | **Reference** |
| --- | --- | --- | --- | --- |
| APAP Uptake Rate | $k_{u}$ | $8.33e-05$ | /s | Approximated value |
| Sulphation Rate | $k_{s}$ | $2.62e+09$ | cell/mol/s | (Reddyhoff et al., 2015) |
| Glucuronidation Rate | $k_{G}$ | $3.46e-05$ | /s | (Reith et al., 2009; Reddyhoff et al., 2015) |
| CYP1A2 Rate | $k_{CYP1A2}$ | $2.60e-06$ | /s | Calculated  value |
| CYP2E1 Rate | $k_{CYP2E1}$ | $2.80e-06$ | /s | Calculated  value |
| CYP3A4 Rate | $k_{CYP3A4}$ | $5.60e-06$ | /s | Calculated  value |
| Reverse Oxidation Rate | $k_{N}$ | $3.65e-07$ | /s | (Reddyhoff et al., 2015) |
| Basal PAPS Synthesis Rate | $b_{S}$ | $3.07e-19$ | cell/mol/s | (Reddyhoff et al., 2015) |
| PAPS Degradation Rate | $d_{S}$ | $2.31e-05$ | /s | (Ookhtens et al., 1985; Aw et al., 1986; Hepatic Glutathione Homeostasis in the Rat: Efflux Accounts for Glutathione Turnover - Lauterburg - 1984 - Hepatology - Wiley Online Library, n.d.) |
| Conjugation rate of NAPQI and GSH | $k_{GSH}$ | $1.85e+13$ | cell/mol/s | (Miner and Kissinger, 1979) |
| NAPQI-Cys Formation Rate | $k_{PSH}$ | $1.27e-03$ | /s | (Reddyhoff et al., 2015) |
| Basal GSH Conjugation Rate | $b_{G}$ | $1.59e-19$ | mol/cell/s | (Remien et al., 2012) |
| GSH Degradation Rate | $d_{G}$ | $2.31e-05$ | /s | (Ookhtens et al., 1985; Aw et al., 1986; Hepatic Glutathione Homeostasis in the Rat: Efflux Accounts for Glutathione Turnover - Lauterburg - 1984 - Hepatology - Wiley Online Library, n.d.) |
| NAPQI-Cys Removal Rate | $d_{C}$ | $3.00e-05$ | /s | Calibrated  value |
| Number of Hepatocytes in Liver | $H_{max}$ | $1.6e+11$ | cells | (Remien et al., 2012) |
| Hepatocyte Regeneration Rate | $r$ | $1.16e-5$ | /s | (Remien et al., 2012) |
| Hepatocyte Damage Rate | $\eta$ | $6.02e+8$ | cell/mol/s | (Remien et al., 2012) |
| Damage Hepatocyte Lyse Rate | $\delta_{Z}$ | $5.79e-05$ | /s | (Remien et al., 2012) |

## Supplementary Figures


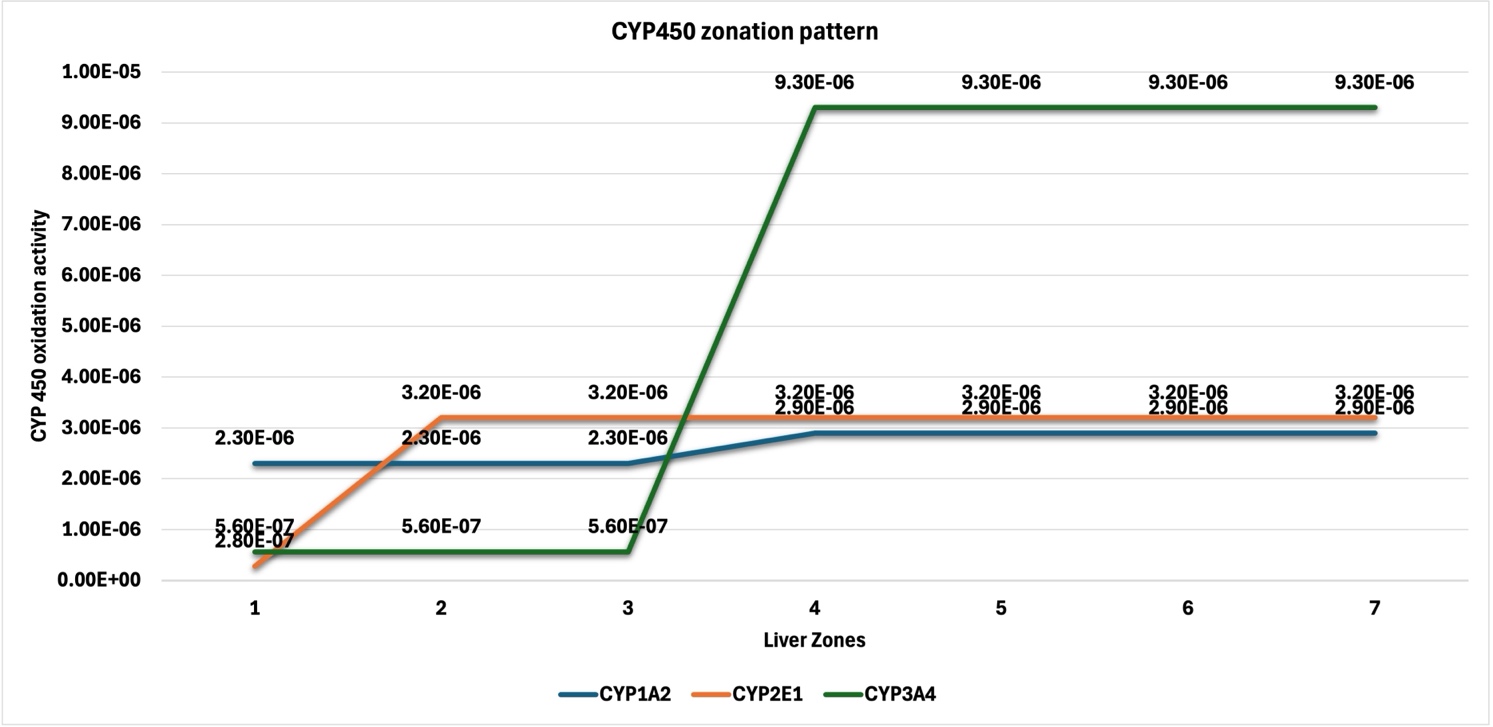


**Supplementary Figure 1: *Zonation distribution of CYP450 isoenzyme (CYP1A2, CYP2E1, CYP3A4) activity***

***Liver zones: 1 (Periportal region) – 7 (Pericentral region)***
